# ‘BREAKS’ Protocol for Breaking Bad News

**DOI:** 10.4103/0973-1075.68401

**Published:** 2010

**Authors:** Vijayakumar Narayanan, Bibek Bista, Cheriyan Koshy

**Affiliations:** Department of Oncology and Palliative Medicine, St. Gregorios Medical Mission Hospital, Parumala, Pathanamthitta, Kerala, India; 1 Department of Anesthesiology, BP Koirala Memorial Cancer Hospital, Bharatpur, Nepal; 2 Department of Pain and Palliative Medicine, Regional Cancer Centre, Thiruvananthapuram, Kerala, India

**Keywords:** Breaking bad news, Communication, Truth disclosure

## Abstract

Information that drastically alters the life world of the patient is termed as bad news. Conveying bad news is a skilled communication, and not at all easy. The amount of truth to be disclosed is subjective. A properly structured and well-orchestrated communication has a positive therapeutic effect. This is a process of negotiation between patient and physician, but physicians often find it difficult due to many reasons. They feel incompetent and are afraid of unleashing a negative reaction from the patient or their relatives. The physician is reminded of his or her own vulnerability to terminal illness, and find themselves powerless over emotional distress. Lack of sufficient training in breaking bad news is a handicap to most physicians and health care workers. Adherence to the principles of client-centered counseling is helpful in attaining this skill. Fundamental insight of the patient is exploited and the bad news is delivered in a structured manner, because the patient is the one who knows what is hurting him most and he is the one who knows how to move forward. Six-step SPIKES protocol is widely used for breaking bad news. In this paper, we put forward another six-step protocol, the BREAKS protocol as a systematic and easy communication strategy for breaking bad news. Development of competence in dealing with difficult situations has positive therapeutic outcome and is a professionally satisfying one.

## INTRODUCTION AND REVIEW OF LITERATURE

Most of the patients appreciate facts about their health. However, the truth telling practices and preferences are a cultural artifact to a certain extent.[1] Honest and truthful disclosure is an extremely difficult task and physicians often find that the disclosure of cancer diagnosis to the patient as one of the most difficult. Very few health care workers have received sufficient training in the “breaking bad news” tactics.[2,3] Bad news is defined as one which is pertaining to situation where there is a feeling of no hope, a threat to a person’s mental or physical well being, a risk of upsetting an established lifestyle or where a message is given which conveys to an individual fewer choices in his or her life.[4] Another definition states “any news that drastically and negatively alters the patient’s view of her or his future” is bad news.[5] Truthful disclosure of psychologically painful information not only hurts the patient and their relatives but also embarrasses the health care worker. A number of empirical studies had documented that physician–patient communication as suboptimal. The main causes for physician’s avoidance of the task of breaking bad news are lack of skills and the reluctance to deal with the patient’s feelings.[6] Physicians and nurses typically miss the full range of concerns of patients with cancer.[7] Breaking bad news is a difficult task and has been extensively studied. Feelings of mistrust, anger, fear, and blame are common reactions if bad news is broken poorly. This communication skill is typically learned through trial and error or observation of senior colleagues.[8] Conveying bad news is more difficult when the clinician has a long-standing relationship with the patient, when the patient is young, or when strong optimism had been expressed for a successful outcome.[9]

The Calman Hine report in 1995 states that “The development of cancer services should be patient centered and should take account of patients’, families’, and carers’ views and preferences, as well as those of the professionals involved in cancer care. Individual perceptions of needs may differ from those of the professionals. Good communication between professionals and patients is especially important.”[10] Despite the availability of an extensive body of research and online resources, communication lacunae pose hurdles in cultivating good therapeutic relationships. One of the most successful approaches in breaking bad news is through client-centered counseling, as proposed by Karl Rogers. He put forward three points in order to achieve a growth producing therapeutic relationship between the client (the patient) and the counselor (the physician). They are (1) be genuine and congruent, (2) offer unconditional positive regard, and (3) feel and communicate a deep, empathetic understanding.[11] According to Rogers, it is the client who knows what is hurting him most and he is the one who knows how to move forward. The fundamental insight of the client is exploited and the bad news is delivered in an orderly manner. A patient-centered communication style has the most positive outcome for recipients of bad news on a cognitive, evaluative, and emotional level.[12] Physician’s attitude and the manner in which he conveys the important news is very crucial for the patient.[13,14] A cool, detached posture of true professionalism would be viewed by patient as well as their relatives as evasive, cold, and unsympathetic at just the time that they are in much need of empathy and support may prove counter productive.[15] Physicians feel ineffective and powerless, when they face a situation that could not be remedied.[16] The physician’s role should not overshadow the patient’s role in hearing and responding to it.[17] The conversation will be more effective if the information given to the patient was summarized as it might have given him an opportunity to correct any misunderstanding.[18] An enquiry into the impact of the illness could throw a better light into the psychosocial aspect of the situation.[19] The doctor should not use any blocking behaviors to immunize himself from the potential distress that he may not be able to handle. Offering premature reassurances and advices before addressing the main concerns of the patient, explaining the distress as normal, playing down the problems, changing the topic of discussion, cracking untimely jokes are some of the blocking behaviors. The six steps SPIKES protocol[17] [Box 1] or ABCDE model[20] [Box 2] seldom offer any chance to miss an issue, though it cannot be followed at all instances.

**Box 1:**
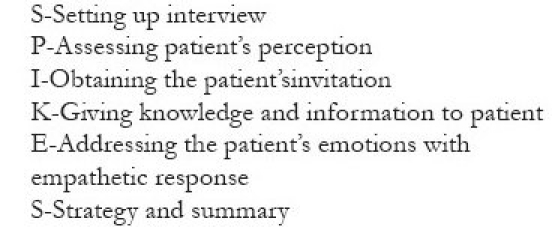
Spikes protocol

**Box 2:**
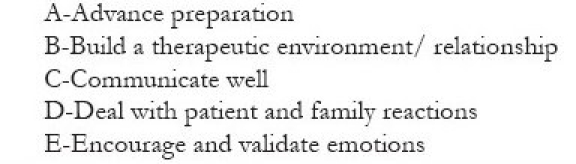
ABCDE model

Keeping eye contact as and when necessary is an important aspect in interpersonal transaction, as it shows interest.[21] Empathy essentially contains an emotional understanding while maintaining sufficient separation so that expert medical skills can be rationally applied to the patient’s problem. The affective, cognitive, and behavioral components of empathy should be understood and allowed to manifest. To 36% of patients, the diagnosis of cancer comes as a complete surprise.[22] A good therapeutic relationship is ensured if the clinician maintains total acceptance and is non-judgmental.[23] Giving information must proceed in an orderly manner, by prioritizing the information needs. A communication that is ambiguous, oblique, or incomplete will leave the patient ill prepared for future and his adjustment reactions will be affected.[24] The authors put forward a rather simple protocol for breaking bad news, ‘BREAKS’ — B –Background, R- Rapport, E – Explore, A –Announce-; K-Kindling and S –Summarize. This mnemonic is very easy to memorize. It can be practiced effectively as well.

### Background

An effective therapeutic communication is dependent on the in-depth knowledge of the patient’s problem. The accessibility of electronic media has given ample scope for obtaining enough data on any issue, though authenticity is questionable. It is highly desirable to prepare answers for all questions that can be anticipated from the patient. The physician must be aware of the patient/relative who comes after “googling” the problem. It may not be possible to answer all questions, but all reasonable doubts of the patient as well as his relatives should be cleared. If the physician has not done his homework meticulously, the session should be postponed. Apart from doing an in-depth study on the patient’s disease status, his emotional status, coping skills, educational level, and support system available are also reviewed before attempting to break the bad news. Cultural and ethnic background of the patient is also very important. The physician has to be sensitive to the cultural orientation of the patient, and it should be respected. The individual’s thinking and actions are governed by his cultural orientation. The physical set up is very important in accomplishing this difficult task. The mobile phone must be switched off. All physical barriers must be removed to maintain eye contact. A co-worker’s help for transcribing the conversation is helpful. Emotional breakdown can be expected; hence, the physician may have to console the patient as well. Regressive behaviors need to be tackled with a complimentary transaction. The appointment length should be sufficient to complete the task.

### Rapport

Building rapport is fundamental to continuous professional relationship. The physician should establish a good rapport with the patient. He needs to have an unconditional positive regard, but has to stay away from the temptation of developing a patronizing attitude. The ease with which the rapport is being built is the key to continue conversation. A hostile attitude has disastrous outcome, so is a hurried manner. It is necessary to provide ample space for the windows of self-disclosure to open up. The patient should be placed in a comfortable position. Present condition of the patient can be enquired through open questions. If the patient is not prepared for the bad news, especially after getting his /her symptoms well palliated, let him finish the reports of well being, and then try to take cues from his conversation to initiate the process of breaking bad news.

### Exploring

Whenever attempting to break the bad news, it is easier for the physician to start from what the patient knows about his/her illness. Most of the patients will be aware of the seriousness of the condition, and some may even know their diagnosis. The physician is then in a position of confirming bad news rather than breaking it. The history, the investigations, the difficulties met in the process etc need to be explored. What he/she thinks about the disease and even the diagnosis itself can be explored, and the potential conflicts between the patient’s beliefs and possible diagnosis can be identified. The dynamics of the family and the coping reservoir of the patient are very important in delivering the bad news. Try to involve the significant other people of the patient in the decision-making process, if allowed by the patient. At least few patients may respond in a bizarre way to the bad news. Hence, a careful exploration of all these points should be carried out. A common tendency from the physicians is that they jump into premature re assurances. Premature reassurance occurs when a physician responds to a patient concern with reassurance before exploring and understanding the concerns.[25] Absolute certainties about longevity cannot be given to a patient. The prognosis can be explained in detail; with all available data. A reasonable conclusion based on the facts can be presented.

### Announce

A warning shot is desirable, so that the news will not explode like a bomb. Euphemisms are welcome, but they should not create confusion. The patient has the right to know the diagnosis, at the same time he has the right to refrain from knowing it. Hence, announcement of diagnosis has to be made after getting consent. The body language of both the physician and patient is very important, and the physician is supposedly a mirror image of the patient. The embarrassment, agony, and fear of the patient should be reflected in the physician (mirroring the emotions), so that the patient will identify the physician as one close to himself. Announcement of the bad news must be in straightforward terms, avoiding the medical jargon completely. Lengthy monolog, elaborate explanations, and stories of patients who had similar plight are not desirable. Information should be given in short, easily comprehensible sentences. A useful rule of thumb is not to give more than three pieces of information at a time.[26]

### Kindling

People listen to their diagnosis differently. They may break down in tears. Some may remain completely silent, some of them try to get up and pace round the room. Sometimes the response will be a denial of reality, as it protects the ego from a potential shatter. A gallows humor is also an expected behavior. These are all predictable responses. Adequate space for the free flow of emotions has to be given. Most of the time, patients will not actively listen to what the physician say after the pronouncement of the status. An overwhelming feeling of a grim fate can ignore further explanations and narratives from the physician’s part. Hence, it is advisable to ensure that the patient listens to what is being told, by asking them questions like “are you there?”, “do you listen to me?” etc. It involves asking the patient to recount what they have understood.

Be clear that the patient did not misunderstand the nature of disease, the gravity of situation, or the realistic course of disease with or without treatment options. While trying to kindle the emotions, care has to be taken not to utter any unrealistic treatment options. The patient and their relatives will cling on to it, and subsequently feel embarrassed because of its unrealistic nature. Answers have to be tailored to the question, and physician should stay away from lecturing to the patient. Lecturing occurs when a physician delivers a large chunk of information without giving the patient a chance to respond or ask questions.[27] Beware of the “differential listening,” as the patient will listen to only those information he/she wants to hear. Dealing with denial is another difficult task. It may be necessary to challenge denial because the patient may have some important unfinished business to conduct, or because the patient is refusing treatment that might alleviate symptoms. In such situations, attempts to break the defense without mutilating the ego should be attempted.

### Summarize

The physician has to summarize the session and the concerns expressed by the patient during the session. It essentially highlights the main points of their transaction. Treatment/care plans for the future has to be put in nutshell. The necessary adjustments that have to be made both emotionally and practically need to be stressed. A written summary is appreciable, as the patients usually take in very little when they are anxious. Offering availability round the clock and encouraging the patient to call for any reasons are very helpful. An optimistic outlook has to be maintained, and volunteer if asked by the patient for disseminating the information to the relatives. The review date also has to be fixed before concluding the session. At the end of the session, make sure that the patient’s safety is ensured once they leave the room. He/she should not be permitted to drive back home all alone, and find whether someone at home can provide support. Patient may even try to commit suicide if he/she feels extremely desperate. Patient should be assured that the physician will be actively participating in all ongoing care plans.

## CONCLUSION

Breaking bad news is part of the art of medicine. A bad news is always a bad news, however well it is said. But the manner in which it is conveyed can have a profound effect on both the recipient (the patient) and the giver (the physician). If done badly, it will hamper the well being of patient, impair the quality of life and future contact with the health care professional will be thwarted. It is a skill that has to be learnt by the physicians and other caregivers and effective methods of communication skills training are available.[28] Lack of proper training will lead to emotional disengagement of the physician from his patients. Good communication has a therapeutic effect on patient and bad communication leads to a detrimental outcome. Communication skills can be improved through structured training programs with appropriate feedback to the trainees. Curricula for teaching the task of breaking bad news include didactic lectures, small-group discussions, role-playing, and teaching in the context of patient care.[29] Role plays and video tapes of the same with constructive suggestions to improve the skills are very much effective. It should be noted that the evidence base of the current practice and training of breaking bad news is not sound. Education and practice in breaking bad news may be ineffective for improving patients’ well-being unless it is informed by a strong evidence base.[30]

We propose this protocol–BREAKS (background, rapport, explore, announce, kindle, summarize) for discussion and further elaboration.
